# Case report: New insights into persistent chronic pelvic pain syndrome with comorbid somatic symptom disorder

**DOI:** 10.3389/fpsyt.2023.1119938

**Published:** 2023-01-20

**Authors:** JiChao Huang, Yi Zhong, Yu Duan, Jie Sun

**Affiliations:** ^1^Affiliated Shuyang Hospital of Nanjing University of Chinese Medicine, Suqian, Jiangsu, China; ^2^NHC Key Laboratory of Mental Health (Peking University), National Clinical Research Center for Mental Disorders (Peking University Sixth Hospital), Peking University Institute of Mental Health, Peking University Sixth Hospital, Peking University, Beijing, China; ^3^Department of Neuroscience, City University of Hong Kong, Hong Kong, Hong Kong SAR, China; ^4^Yangzhou University, Yangzhou, China; ^5^Pain Medicine Center, Peking University Third Hospital, Beijing, China

**Keywords:** chronic pelvic pain syndrome, comorbidities, somatic symptom disorder, COVID-19, case report

## Abstract

Chronic pelvic pain syndrome (CPPS) is generally defined as pain in the pelvic area that persisted for 3–6 months or longer. The pain can be constant or episodic and functionally disabling. Any dysfunction of the central nervous system can lead to central sensitization, which enhances and maintains pain as well as other symptoms that are mediated by the central nervous system. It occurs in subgroups of nearly every chronic pain condition and is characterized by multifocal pain and co-occurring somatic symptoms. Somatic symptom disorder (SSD) is defined as a condition in which having one or more somatic symptoms, such as excessive worries, pressure, and catastrophic events. These symptoms can be very disruptive to a patient’s life and can cause significant distress. SSD cases with severe symptoms frequently undergo repeated medical investigations and the symptoms often lead patients to seek emergency medical treatment and consult with specialists repeatedly, which is a source of frustration for patients and clinicians. Here we report a case that Asian female with persistent CPPS with comorbid SSD, who got in trouble for up to 8 years. This case reminds clinicians to pay excessive attention to the diagnosis of CPPS with comorbid SSD after recovery from acute COVID-19, with hope of raising awareness in the identification of SSD and present new insight into appropriate treatment for each woman who suffers from it.

## Introduction

Chronic pelvic pain syndrome (CPPS) is generally defined as pain in the pelvic area that persisted for 3–6 months or longer. The pain can be constant or episodic and functionally disabling (1). Approximately one-third of women with CPPS seek medical treatment, although the prevalence of the disease ranges from 4 to 16% (2). The prevalence of female CPPS varies globally according to the inclusion criteria, but is estimated to affect 6–25 percent of women of reproductive age (3–5). It was estimated that CPPS treatment costs 880 million dollars annually (6). Women reported losing working days about 15% of the time, and work efficiency decreased about 45% of the time (7, 8).

In addition to central sensitization of pain, CPPS is a symptom of pathology in other somatic structures or viscera (9). However, the cause of pain may not be identified in some women, and some women will experience persistent pain despite being treated for presumed causes (10). A variety of factors can contribute to the development of CPPS, including pathology or dysfunction of any of the multiple organ systems at play in the pelvis (9). In recent years, Somatic symptom disorder (SSD) has become increasingly prevalent among adolescents (11, 12). SSD are defined by the DSM-5 as a condition in which having one or more somatic symptoms, as well as excessive worries, and as spending too much time and energy dealing with them, resulting in a loss of social and personal opportunities (13, 14). People with this disorder may have symptoms such as chronic pain, fatigue, dizziness, or shortness of breath. These symptoms can be very disruptive to a person’s life and can cause significant distress.

The misdiagnosis of complex, unusual, and multisystem diseases is common. The pursuit of curing a symptom without a physical cause could drain hospital and patient resources (15). Herein, we discuss an Asian female with persistent CPPS with SSD, who got in trouble for up to 8 years and present new insight about CPPS with SSD.

## Case report

An Asian female, Miss A, 23 years old, presented with persistent chronic pelvic pain disorder pain. As of 2014, she was initially experiencing irregular lumborum pain, feeling cold, and gradually developing lumbo-abdominal dull pain without any previous psychiatric history. The sudden onset of dysmenorrhea is more than 10 days before menstrual period and relieved by taking Yasmin. However, after taking Yasmin for half a year, she became depressed, irritable, and cried easily. The pain in the pelvis intensified in 2021, accompanied by swelling in the vulvar area as well as pain in the pubic region (Table 1). Her depression history warranted a referral to the psychiatry team after interventions failed. It took her 4 months to be admitted to the psychiatry department outside a hospital in April 2022. The clinician adjusts Sodium Valproate and Lorazepam due to rapid mood changes into anger or depression with persistently elevated moods and increased talking activity. The physical examinations at several hospitals revealed no abnormalities.

**TABLE 1:** Characteristics of pain from 2014 to 2021.

Year	2014	2016	2021	April–August 2022
Characteristics of pain	Lumborum pain irregularly		Lumbo-abdominal pain intensified	Lumbo-abdominal pain intensified
	Lumbo-abdominal dull pain	Lumbo-abdominal dull pain irregularly	Accompanied with the vulvar and pubes swelling pain	With the vulvar and pubes swelling pain
	Dysmenorrhea	Dysmenorrhea	Dysmenorrhea	Dysmenorrhea

It was diagnosed that SSD was present on 14 August 2022, and treatment to target SSD was begun the following day. In the wake of her somatic symptoms becoming more severe and her hospital course becoming more refractory, we transferred her to our inpatient psychiatry ward. She is being treated on this ward for the remainder of her stay. According to the psychiatric examination conducted on admission, she had persistent pelvic pain, which became worse when she walked or became tired. In a curious turn of events, the pain disappeared once she fell asleep. The clinician adjusted the doses of several medications, including Vortioxetine (10 mg qd), Tandospirone (30 mg qd), Pregabalin (75 mg qd), Trazodone (25 mg qd), Fluphenazine (2 mg qd), and Methycobal (1.5 mg). The patient’s basic metabolic panel, white blood cell count, blood cultures, thyroid function, autoimmune tests, cerebrospinal fluid studies, CT scan of the head, and MRI of the brain were all normal.

In the following weeks, Vortioxetine and Pregabalin doses were increased to 15 mg each and 150 mg, respectively (Table 2). Her insomnia and somatic symptoms were hoped to be diminished by trials of transcranial magnetic stimulation (TMS), transcranial direct-current stimulation (tDCS) and psychotherapy. Following the adoption of the adjusted therapy, Miss A reported that her pain had eased and showed evidence of improvement. The SSD strategies worked well for her, and her pain diminished. A follow-up appointment is scheduled for her to consider further therapy after she had been discharged home. During our phone contact, she followed up for 2 months, but was unwilling to follow up afterward.

**TABLE 2:** Prescribed medication over 8 years.

Year	2014	2016	2021	April 2022	June 2022	3 August 2022	22 August 2022	30 August 2022
**Antidepressants**								
Duloxetine	–	–	–	1# qd	–	–	–	–
Trazodone	–	–	–	1# qn	1# qn	25 mg qd	25 mg qd	25 mg qd
Brintellix	–	–	–	–	–	10 mg qd	15 mg qd	20 mg qd
**Anxiolytics**								
Tandospirone	–	–	–	3# qd	–	30 mg qd	30 mg qd	60 mg qd
Pregabalin	–	–	–	–	–	75 mg qd	150 mg qd	150 mg qd
**Antipsychotics**								
Sodium valproate	–	–	–	1# qd	–	–	–	–
Fluphenazine	–	–	–	–	4# qn	2 mg qd	–	–
Paliperidone	–	–	–	–	–		3 mg qd	3 mg qd
**Sedatives**								
Lorazepam	–	–	–	0.5# qd	–	–	–	–
Zolpidem	–	–	–	1# qn	–	–	–	–
**Others**								
Methycobal	–	–	–	–	–	1.5 mg qd	1.5 mg qd	1.5 mg qd
Vitamin B6	–	–	1# tid	1# tid	1# tid	1# tid	1# tid	1# tid
Yasmin	*	*	–		–	–	–	–

*Take the medicine but no record the accurate dosage.

## Discussion

Chronic pelvic pain syndrome is a painful condition that may be a result of disease in a somatic or visceral structure, or a manifestation of central sensitization to pain (16). Chronic pain may be the only diagnosis available for some women who suffer from CPPS, and the condition can be frustrating for both patients and clinicians (4). It is likely that central sensitization contributes to chronic pelvic pain syndrome in these women. Any dysfunction of the central nervous system can lead to central sensitization, which enhances and maintains pain as well as other symptoms that are mediated by the central nervous system (17). It occurs in subgroups of nearly every chronic pain condition and is characterized by multifocal pain and co-occurring somatic symptoms (18).

The use of devices unjustified by a clinical diagnosis may result in a real disability for the patient suffering from somatic symptom disorder (SSD) (19). Multiple factors can contribute to SSD, including excessive pressure, catastrophic events including abuse or bullying, chronic diseases, mental illness, and a family history of chronic pain (20). SSD cases with severe symptoms frequently undergo repeated medical investigations, a characteristic that stands out as particularly relevant and common (13). The aforementioned symptoms often lead patients to seek emergency medical treatment and consult with specialists repeatedly (21). Hospital admissions are also common and extensive investigations are conducted repeatedly.

The cost of medical care for people with SSD is high, accounting for about $250 billion in incremental costs in the USA alone each year (22, 23). SSD is associated with a number of psychosocial factors, including genetics, sexual abuse, cognitive distortions, and family conflict. Global public health has been greatly impacted by the COVID-19 pandemic. It is crucial to recognize that mental health issues can be influenced by various stressors, such as fears of infection, social distancing measures, and economic and social burdens. Studies have shown that individuals may be at risk of developing SSD after being infected with COVID-19 (24–27). Primary care clinicians are generally effective in managing these patients. Diagnoses may vary, with a lack of response to various treatment attempts. Multiple treatments may not be effective, depending on the diagnosis. The vicious circle of persistent SSD symptoms, seeking medical care, and ineffective treatment can negatively influence the patient and his or her family, with time spent searching for possible causes and, in extreme cases, disrupting the family dynamics.

Children and adolescents with SSD may suffer adverse effects if a diagnosis of SSD is missed in childhood or adolescence, or invasive therapies are prolonged. When SSD is not recognized, school attendance can be lost, functional disability might be permanent, and psychiatric diseases, such as depression or anxiety disorder, might go unnoticed. Additionally, patients with SSD frequently had comorbid psychiatric conditions, including anxiety, depression, and post-traumatic stress disorder. There is a strong correlation between psychiatric disorders and persistent pain, which can reduce adherence to treatment and lead to self-medication or self-treatment with drugs (28). The majority of adolescents with SSD had neurologic symptoms, while 39% had pain-related symptoms (29). In studies of patients with SSD with depression and anxiety disorders, high comorbidity rates were observed (30–32). Approximately 30–60 percent of primary care SSD patients suffer from comorbid anxiety disorder and/or depression disorder. A serious concern is that patients suffering from SSD have a higher risk of suicide.

According to the biopsychosocial framework, biologic, psychological, and social factors are dynamically intertwined in pain. In terms of chronic pain treatment, cognitive behavioral therapy (CBT) is a first-line option, and more research has been done on CBT than other kinds of psychotherapy, which is why we recommend it (33, 34). Psychological approaches such as motivational interviewing, mindfulness, meditation, and other relaxation techniques can be used to treat SSD. Furthermore, neuromodulation, which modulates relevant brain networks, may be a promising treatment option. In a systematic review, TMS was found to be the most frequently used treatment, followed by electroconvulsive therapy and tDCS (35). It may reduce opioid use when combined with exercise and other components of interdisciplinary rehabilitation. No matter whether patients with SSD are diagnosed with known overlapping pelvic pain conditions, multimodal treatment may provide benefit to patients without SSD that fails to respond to syndrome-specific treatment.

## Conclusion

Women find it difficult to accept the diagnosis of CPPS with comorbid SSD, despite the availability of adequate multidisciplinary hospital services. In addition to being common, it is associated with high levels of suffering. Parent and physician often seem to do nothing when they are asked to do nothing as part of their treatment. This case reminds clinicians to pay excessive attention to the diagnosis of CPPS with comorbid SSD after recovery from acute COVID-19, with hope of raising awareness in the identification of SSD and present new insight into appropriate treatment for each woman who suffers from it.

## Data availability statement

The original contributions presented in this study are included in the article/supplementary material, further inquiries can be directed to the corresponding author.

## Ethics statement

The studies involving human participants were reviewed and approved by the Peking University Sixth Hospital. The patients/participants provided their written informed consent to participate in this study.

## Author contributions

YZ and JH drafted the manuscript. JS and YD critically revised the manuscript. All authors read and approved the final manuscript.
